# Overcoming the problem of heterologous proteins folding to improve the efficiency of yeast bioproducers

**DOI:** 10.18699/vjgb-25-140

**Published:** 2025-12

**Authors:** N.V. Dorogova, S.A. Fedorova

**Affiliations:** Institute of Cytology and Genetics of the Siberian Branch of the Russian Academy of Sciences, Novosibirsk, Russia; Institute of Cytology and Genetics of the Siberian Branch of the Russian Academy of Sciences, Novosibirsk, Russia

**Keywords:** yeast bioproducers, protein folding, endoplasmic reticulum, molecular chaperones, recombinant proteins, дрожжи-продуценты, фолдинг белков, эндоплазматический ретикулум, молекулярные шапероны, рекомбинантные белки

## Abstract

In the last few decades, yeasts have been successfully engineered to be an excellent microbial cell factory for producing recombinant proteins with desired properties. This was due to their cost-effective characteristics and the successful application of genomic modification technologies. In addition, yeasts have a conserved post-translational modification pathway among eukaryotic organisms, which ensures the correct folding of recombinant proteins. However, the folding machinery cannot always cope with the load caused by the overexpression of recombinant genes, leading to the accumulation of misfolded proteins, the formation of aggregates and low production. Therefore, the protein-folding capacity of the endoplasmic reticulum (ER) remains one of the main limitations for heterologous protein production in yeast host organisms. However, thanks to many years of effective research of the fundamental mechanisms of protein folding, these limitations have been largely overcome. The study of folding in both model organisms and bioproducers has allowed to identify the molecular factors and cellular mechanisms that determine how a nascent polypeptide chain acquires its three-dimensional functional structure. This knowledge has become the basis for developing new effective techniques for engineering highly productive yeast strains. In this review, we examined the main cellular mechanisms associated with protein folding, such as ER transition, chaperone binding, oxidative folding, glycosylation, protein quality control. We discuss the effectiveness of applying this knowledge to the development of various engineering techniques aimed at overcoming bottlenecks in the protein folding system. In particular, selection of optimal signal peptides, co-expression with chaperones and foldases, modification of protein quality control, inhibition of proteolysis, and other techniques have allowed to enhance the ability of yeast bioproducers to effectively secrete heterologous proteins.

## Abstract (ru)

За последние несколько десятилетий дрожжи стали наиболее эффективными биопродуцентами рекомбинантных белков с различными потребительскими свойствами. Это стало возможным благодаря их экономически выгодным характеристикам и успешному применению генно-инженерных технологий. Кроме того, дрожжи обладают консервативным для эукариотических организмов механизмом посттрансляционной модификации белков, который обеспечивает их корректный фолдинг, необходимый для дальнейшей секреции и функциональной активности. Однако аппарат фолдинга не всегда справляется с нагрузкой, вызванной сверхэкспрессией рекомбинантных генов, что приводит к накоплению неправильно свернутых белков, образованию агрегатов и низкой продуктивности дрожжевых штаммов. Таким образом, способность к фолдингу белков в эндоплазматическом ретикулуме по-прежнему остается одним из основных ограничений при синтезе рекомбинатных белков в дрожжевых клетках. Эти ограничения были в значительной степени преодолены благодаря многолетним эффективным исследованиям фундаментальных механизмов белкового фолдинга. Изучение фолдинга как у модельных организмов, так и у биопродуцентов позволило выявить молекулярные факторы и клеточные механизмы, определяющие формирование трехмерной функциональной структуры растущей пептидной цепи. Полученные знания легли в основу разработки новых эффективных методов конструирования высокопродуктивных штаммов дрожжей. В данном обзоре мы рассмотрели основные клеточные механизмы, связанные с фолдингом белков, такие как транспорт через эндоплазматический ретикулум, взаимодействие с шаперонами, окислительный фолдинг, гликозилирование и контроль качества белков. Мы обсудили эффективность применения этих знаний при разработке различных инженерных методов, направленных на преодоление узких мест в системе белкового фолдинга. В частности, подбор оптимальных сигнальных пептидов, коэкспрессия с шаперонами и фолдазами, модификация клеточных механизмов контроля качества белков, ингибирование протеолиза и некоторые другие приемы позволили повысить возможности использования дрожжей-продуцентов в качестве эффективной производительной платформы для экспрессии и секреции рекомбинантных белков

## Introduction

Yeast expression systems are excellent for the production of valuable recombinant proteins and peptides widely used as biopharmaceuticals and industrial enzymes. They are commercially viable bioproducers due to their high growth rate, resistance to harmful microbiota, ability to assimilate many food sources, and fairly easy to cultivate in industrial conditions (Thak et al., 2020; Madhavan et al., 2021; De Brabander et al., 2023). The development of genetic and metabolic engineering has increased the efficiency of yeast strains, mainly due to the use of genomic technologies: strong promoters, new vector elements with improved inducers and enhancers, targeted mutagenesis, signaling molecules, high-performance devices for cloning, screening and fermentation (De Brabander et al., 2023; Tsuda, Nonaka, 2024). However, expression at the transcriptional and translational levels often does not correlate with the level of secretion of heterologous proteins, which is due to the insufficient efficiency of the folding mechanism (Ishiwata-Kimata, Kimata, 2023; Zahrl et al., 2023). Therefore, the search for new technological methods for optimizing the synthesis and increasing the yield of heterologous proteins in yeast cells remains an urgent task. A significant direction for its solution is overcoming the problem of folding target proteins into the correct three-dimensional structure

Proper folding is necessary for the functional activity of synthesized proteins, their intracellular transport and further secretion. Recombinant proteins are secretory and go through a secretory pathway, beginning with folding in the ER and ending with release into the extracellular environment (culture media) (Raschmanová et al., 2021). Proteins are translocated to the ER in an unfolded state and then undergo modification and folding involving ER-resident chaperones, folding enzymes, and glycosylation (Hartl et al., 2011; Saibil, 2013). Disruptions in this machinery result in the accumulation of misfolded proteins in the cytoplasm, where they are recognized by the Protein Quality Control (PQC) system that regulates cellular homeostasis (Korennykh, Walter, 2012). This system includes the Unfolded Protein Response (UPR) signaling pathway, which can trigger refolding of misfolded proteins or initiate their proteolysis

In some cases, misfolded proteins clump together to form aggregates or Inclusion Bodies (IBs), which can cause cell damage (Yamaguchi, Miyazaki, 2014). Such IBs often contain potentially active proteins with a normal secondary structure, which can be recovered from these aggregates under appropriate conditions (Burgess, 2009; Yamaguchi, Miyazaki, 2014; Singhvi et al., 2021). However, their isolation and refolding procedures are complex, expensive, and inefficient (Singhvi et al., 2021). It is therefore clear that the solution to the folding problem of heterologous proteins must be directly linked to their production process: protein folding engineering and quality control in yeast host strains. In this article, we review various cellular mechanisms and signaling pathways that influence heterologous protein folding and discuss the latest updates to biotechnological strategy allowing to address this issue in order to maximize the yield of recombinant proteins

## Post-translational modifications and folding

**Preparation for folding**

In yeast, as in other eukaryotes, a newly synthesized peptide must undergo post-translational modification and folding in the endoplasmic reticulum to form the correct spatial conformation. A stable 3D structure determines the functional activity of the protein and its subsequent traffic through the secretory pathway (Schwarz, Blower, 2016).

Translation of secretory proteins occurs on cytosolic ribosomes and they are then co-translationally or post-translationally directed to the ER by a specific protein-RNA complex – Signal Recognition Particle (SRP). SRP binds to the N-terminal sequence of a precursor protein, which is called the signal peptide (SP). Transfer across the ER membrane occurs in an unfolded state of the nascent protein and is dependent on the Sec water channel, a multiprotein complex that spans the membrane (Berner et al., 2018; O’Keefe et al., 2022) (see the Figure).

**Fig. 1.**
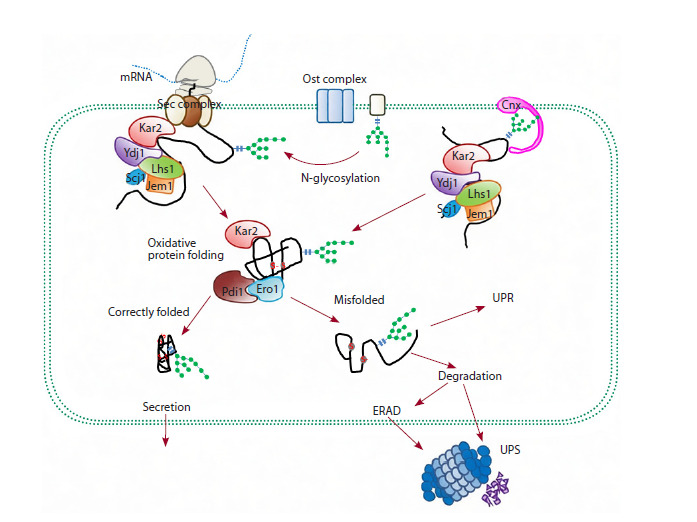
Nascent secretory protein is transferred co-translationally into the ER lumen through the Sec multiprotein complex that spans the ER membrane. At the ER membrane, the protein undergoes N-glycosylation involving the OST complex. The N-glycan-modified peptide chain binds to calnexin (Cnx). When the unfolded nascent chain appears in the ER lumen, it interacts with Kar2 and Ydj1. Jem1, Scj1 and Lhs1 assist in this process. In the ER lumen, the nascent peptide undergoes an OPF process involving Pdi and Ero, which results in disulfide bonds. Accumulation of misfolded proteins in the cytoplasm induces the UPR response which can trigger refolding of misfolded proteins or initiate ERAD

When the unfolded nascent chain appears in the ER lumen, its hydrophobic sequence elements are recognized by ER-resident HSP chaperones: Kar2 (yeast Hsp70) and Ydj1 (yeast Hsp40) (Braakman, Hebert, 2013; Hendershot et al., 2024). They bind to the hydrophobic amino acid side chains exposed by the unfolded proteins. There is evidence that Kar2 assists in the folding of nascent proteins as they enter the ER and remains bound until folding is complete. The Ydj1 chaperone forms transient complexes with Kar2, facilitating its binding to non-native polypeptides (see the Figure). Thus, chaperones prevent premature misfolding of the immature polypeptide chain and protect it from aggregation (Omkar et al., 2024; Ruger-Herreros et al., 2024).

According to some data, the chaperone function of Kar2 in these events also depends on members of the DnaJ-like protein family, such as Jem1 and Scj1, and the nucleotide exchange factor Lhs1 (see the Figure). These co-chaperones promote the ATPase cycle and thus maintain Kar2 activity (Schlenstedt et al., 1995; Steel et al., 2004; de Keyzer et al., 2009).

**Oxidative protein folding**

3D structure formation begins with the process called Oxidative Protein Folding (OPF), which results in the formation of disulfide bonds due to the oxidation of thiol groups of cysteine (Hatahet, Ruddock, 2009; Palma et al., 2023). OPF is carried out by Protein disulfide isomerase (Pdi) (see the Figure). Pdi not only is responsible for the formation of the disulfide bonds in unfolded eukaryotic proteins, but also catalyzes the rearrangement of incorrect disulfide bonds (isomerase activity) (Gross et al., 2006). The OPF pathway, like Pdi, is conserved across eukaryotic organisms. Yeast Pdi1 protein is encoded by the pdi1 gene, and pdi1-deleted yeast strains have low viability and accumulate secretory proteins within the ER lamellae (Mizunaga et al., 1990; Frand, Kaiser, 1998).

The transition of Pdi to the active form is catalyzed by ER sulfhydryl oxidase 1 (Ero1). Ero1 is an oxidoreductase that oxidizes Pdi1 via direct thiol-disulfide exchange (conversion of cystine to cysteine) (Gross et al., 2006; Sevier, Kaiser, 2006). Yeast contains a single ero1 gene that encodes the Ero1 protein. The loss of ero1 is lethal for yeast (Niu et al., 2016). Thus, Pdi and Ero act synergistically; for the correct catalysis of disulfide bonds in proteins, a balance of these two factors is necessary (Niu et al., 2016; Wang L., Wang C.C., 2023). The need to maintain such a balance is also due to the fact that the activation of Pdi and Ero occurs via the oxidation–reduction type and the high rate of disulfide bond formation in cells and tissues should create dangerous levels of oxidative stress (Gasser et al., 2008).

**Role of glycosylation in promoting protein folding**

Proper folding of most proteins requires post-translational modification known as glycosylation. In yeast, secretory proteins are glycoproteins and they contain covalently linked oligosaccharides, which are mainly mannose residues (see the Figure). Predominantly, yeast polypeptides are N- glycosylated, i. e. mannose is N-glycosidically linked to the β-amido group of asparagine. This reaction is catalyzed by the Oligosaccharyltransferase Enzyme Complex (OST) (Kelleher, Gilmore, 2006) and occurs on ER membranes. OST transfers Glc3Man9GlcNAc2-oligosaccharide (where Glc is glucose, Man is mannose, and GlcNAc is N-acetylglucosamine) from the lipid-pyrophosphate donor, dolichol diphosphate, to asparagine residues of nascent polypeptide chains. The proteinlinked oligosaccharide is called N-glycan. This glycan has maintained a well-conserved structure throughout evolution and is characteristic of all eukaryotes (Qi et al., 2020). Glycans undergo further processing in the ER by glycosidases. In Saccharomyces cerevisiae, the following glycosidases have been described: alpha-glucosidase I encoded by CWH41, alpha-glucosidase II encoded by ROT2, alpha1,2-manno-sidase encoded by MNS1. Glycosidases partially deglycosylate and shorten the N-glycan (Herscovics, 1999; Lehle et al., 2006)

Glycosylation promotes folding by enhancing the solubility and stability of the proteins in the ER and the Golgi. Oligosaccharide residues are a marker for interactions with certain chaperones, also assisting in the folding of glycoproteins (Parodi, 2000; Xu C., Ng, 2015).

The N-glycan-modified polypeptide chain is recognized by lectin chaperones (Ware et al., 1995; Caramelo, Parodi, 2015). In mammalian cells, two related ER lectin chaperones, calnexin (Cnx) and calreticulin (Crt), are important for the proper folding of newly synthesized glycoproteins. Calnexin is an integral membrane protein, and calreticulin is a soluble protein found in the ER lumen (see the Figure). They retain glycoproteins in the ER during translation by inhibiting their aggregation and formation of non-canonical disulfide bridges, and also promote their association with other chaperones. In yeast (S. cerevisiae), only the calnexin homologue Cne1 has been identified. Cne1 is structurally similar to mammalian calnexin except that it lacks a cytoplasmic tail and does not bind calcium (Parlati et al., 1995). Yeast calnexin has been shown to function as a molecular chaperone similar to mammalian calnexin (Xu X. et al., 2004). A calnexin-like transmembrane protein has also been identified in S. pombe (Núñez et al., 2015). However, genes encoding a calreticulin homologue have not yet been identified in the yeast genome

## Protein Quality Control

All eukaryotes have conserved mechanisms that control cellular proteostasis and protect the cell from stress. These are the three main pathways that comprise the quality control system: UPR, ERAD (ER-associated degradation), and autophagy. There are essentially two alternative cellular responses to the accumulation of abnormal proteins. UPR promotes their refolding repair through additional activation of ER chaperones and folding enzymes, while ERAD and autophagy target them for degradation (see the Figure).

**Unfolded protein response**

In eukaryotes, UPR includes ER-localized molecular chaperones that participate in sensing misfolded proteins, activating downstream signaling cascades, and mitigating proteotoxic ER stress: Inositol-requiring enzyme 1 (Ire1), Activating transcription factor 6 (Atf6), and Protein kinase R-like ER kinase (PERK). However, only Ire1 is highly conserved and has been found in unicellular eukaryotes, including yeast (Schroder et al., 2003; Mori K., 2022). Presumably, through its luminal domain, Ire1 enables direct interaction with exposed hydrophobic groups of misfolded proteins in the ER. This reaction results in the activation of Ire1, and as a result, its C-terminal RNase domain mediates splicing of the Hac1 gene mRNA removing a 252-nucleotide intron near the 3′ end. Then Rlg1, a tRNA ligase, ligates the hac1 transcript cleaved by Ire1p. Its spliced and unspliced forms are termed Hac1i and Hac1u, respectively (“i” and “u” for induced and uninduced) (Schroder et al., 2003; Xia, 2019). Hac1i mRNA is translated into the transcription factor Hac1. It is then transported into the nucleus where it induces transcription of a large number of genes involved in the UPR mechanism (Hernández-Elvira et al., 2018) including those encoding ER-located molecular chaperones and protein modification enzymes such as kar2, pdi1, ero1, ecj1, lhs1, jem1. Additionally, the Ire1/Hac1 pathway is essential for activating genes that carry out ERAD functions promoting selective removal of terminally damaged proteins (Friedlander et al., 2000; Travers et al., 2000).

Hac1 orthologs have been identified in various yeast species, such as Pichia pastoris, Hansenula polymorpha, Kluyveromyces lactis, Yarrowia lipolytica, Candida albicans, and Candida parapsilosis. All of these species share the Ire1- dependent mechanism of splicing the Hac1 transcripts in response to ER stress (Hernández-Elvira et al., 2018; Fauzee et al., 2020; Ishiwata-Kimata, Kimata, 2023).

However, it has been reported that S. pombe probably does not contain a Hac1 ortholog. Its Ire1 triggers a process called Regulated Ire-dependent decay (Kimmig et al., 2012). In S. pombe, stress-activated IRE1 cleaves mRNAs located in the ER, leading to their exonuclease-mediated degradation (Hernández-Elvira et al., 2018).

**ER-associated degradation**

Proteins that have not achieved their native conformation after repeated folding remain in the ER, bound to ER chaperones that prevent their aggregation. If a protein remains unfolded in the ER for too long, it is identified as potentially harmful and eliminated via ER-associated degradation. The process involves several steps: the recognition of substrates in the lumen and membrane of the ER, their translocation into the cytosol, ubiquitination and degradation in the 26S proteasome. Thus, ERAD is associated with the highly conserved ubiquitin proteasome system (UPS) (Ruggiano et al., 2014; Krshnan et al., 2022). In all eukaryotes, it includes the following main components: ubiquitin-activating enzyme (E1), ubiquitin-conjugating enzyme (E2), ubiquitin ligase (E3), and 26S proteasome (Pickart, 2001). If a misfolded glycoprotein stays in the ER for a critically long time, its N-linked glycans are trimmed by yeast 1,2-mannosidase1 – Htm1. This trimmed N-glycan is recognized by a lectin chaperone known as Yos9. At the same time, the Hrd3 protein (HMG-CoA reductase degradation protein 3) associates with hydrophobic amino acid residues exposed on the surface of misfolded glycoproteins (Thibault, Ng, 2012; Berner et al., 2018). After recognition and binding to Hrd3 and Yos9, the substrate protein is transferred to the E3 ubiquitin ligases complex responsible for ubiquitination – Hrd1 (HMG-CoA reductase degradation protein 1). Hrd1 is embedded in the ER membrane and is in conjunction with Der1 (Degradation in the endoplasmic reticulum protein 1) and Usa 1 (U1 SNP1-associating protein 1), which mediates retrotranslocation of misfolded proteins through the ER membrane from the lumen side of the cytosolic membrane. The Hrd1 E3 ubiquitin ligase contains a RING domain that accepts ubiquitin from the membrane-associated protein Ubc7 (E2 ubiquitin- conjugating ligase). Finally, Hrd1 transfers ubiquitin to the substrate protein. Proteins covalently linked to one or more ubiquitin molecules are recognized by proteasome (Preston, Brodsky, 2017; Berner et al., 2018; Krshnan et al., 2022).

## Ways to solve the protein folding problem in yeast synthetic biology

**Selection of ER-targeting signal peptides**

ER-targeting signal peptides (SPs) are critical components for the secretion of heterologous proteins because they are required for their correct transport and localization to the ER, where modification and folding occur (Zha et al., 2023). SPs are short sequences, 15 to 30 amino acids in length, mostly located at the N-terminus of the secreted proteins. SPs typically consist of a series of hydrophobic amino acids (core region) that embed into membranes, positively charged residues at the N-terminus (necessary for proper topology of the polypeptide), and a cleavage site at the C-terminus. When an SP is cleaved by a signal peptidase, it releases the secreted protein into the ER lumen (Zha et al., 2023).

Recent research has revealed that the secretion efficiency of heterologous proteins is strongly dependent on their successful combination with SPs. Therefore, various bioinformatic and experimental studies have been carried out to elucidate the optimal SP sequence, allowing to maximize the efficiency of protein secretion.

A wide range of signal sequences, both from native genes (including those from different organisms) and synthetic molecules, are used to improve heterologous expression in yeast. For example, in Komagataella phaffii (P. pastoris), a widespread host microorganism, the most commonly used secretion signal is the mating pheromone of the α-factor (MFα1) from S. cerevisiae (Eskandari et al., 2023). Although this SP has proven its effectiveness in increasing the yield of heterologous proteins, work continues on its modification and the search for more productive variants. In particular, it was shown that some single amino acid substitutions of the MFα signal sequences provided a significant increase in the production of secreted proteins (Ito et al., 2022). An improved secretion signal was also obtained by creating a chimeric construct combining the MFα1 leader region and the Ost1 signal sequence. This hybrid variant turned out to be more effective compared to the original MFα1 SP (Barrero et al., 2018). The emergence of new improved modifications of MFα1 SPs was facilitated by the analysis of mutations accumulated in MFα signal sequences during yeast evolution. This allowed to identify specific motifs as well as their combinations (mutation synergism) that are important for enhancing yeast enzyme secretion (Aza et al., 2021). Promising results were obtained by combining bioinformatic prediction of the efficiency of certain signal sequences and subsequent experimental validation. For example, Duan and colleagues (2019) discovered four new endogenous signal peptides, including Dan4, Gas1, Msb2, and Fre2, according to the reported secretome and genome of P. pastoris. Their properties were investigated experimentally using three reporter proteins, and these SPs were shown to be superior to α-MFs in the production of heterologous proteins (Duan et al., 2019).

The SignalP databases (www.signalpeptide.de) can be used as a resource for selecting suitable SPs. Mori and colleagues (2015) created a library of 60 S. cerevisiae SPs that were identified in SignalP 3.0 using SOSUI software. The authors experimentally showed that six those SPs can maximize secretion of heterologous proteins (Mori A. et al., 2015).

While some progress has been made in bioinformatically predicting the efficiency of particular signal peptides for recombinant proteins, their potential has only been explored in laboratory settings. There is no reliable information yet on their successful application in biotech platforms, and it is unclear how they will function in specific yeast strains and in combination with specific target proteins.

**Increased activity of the ER folding network**

*Positive effects of chaperone addition*

Traditionally (since the 90s), yeast strain engineering has used the method of simultaneous expression (co-expression) of genes encoding heterologous proteins and genes encoding chaperones and folding enzymes. It has been shown for a variety of heterologous proteins that the introduction of extra copies of these genes into yeast host cells increases the secretion and decreases the aggregation of recombinant proteins. For example, Robinson et al. (1994) demonstrated that overexpression of Pdi in S. cerevisiae led to a four- to tenfold increase in secretion yields of human protein. Shusta and coauthors (1998) reported 2–6-fold increased secretion titers for single-chain antibody fragments upon co-overexpression of the Kar2 chaperone or Pdi. Simultaneous overexpression of Pdi and Kar2 resulted in a synergistic up to eightfold increase (Shusta et al., 1998).

The addition of Pdi and Kar2, both together and separately, increased the yield of recombinant proteins used for medical purposes: antithrombotic factor, hirudin (Kim et al., 2003); mammalian peptide recognition proteins (Yang et., 2016); Necator americanus secretory protein (Inan et al., 2007); fragment of single-chain antibody A33 (Damasceno et al., 2007); hydrophobins (Sallada et al., 2019), virus glycoprotein, RABV-G (Ben Azoun et al., 2016).

In some cases, chaperone activity is enhanced by their co-expression with molecular partners and cofactors. For example, Kar2 functionality depends on the ATPase cycle, which is promoted by the Jem1 co-chaperone and the Lhs1p nucleotide exchange factor (Steel et al., 2004). Joint expression of genes encoding these factors has been shown to stimulate Kar2 activity and increase production of recombinant human proteins (Payne et al., 2008).

As we wrote above, yeast strains overexpressing Pdi are the most frequently used in the production of correctly folded and functionally secreted recombinant proteins. However, in the oxidative protein folding pathway, Pdi acts in partnership with Ero1-oxidase. Therefore, co-overexpression of these genes can improve the efficiency of protein folding and secretion. Beal and colleagues (2019) developed a new methodology enabling the quantitative assessment of the interaction of Pdi1 and Ero1, and based on it provided a platform for the design of more efficient heterologous protein expression systems in yeast. The high efficiency of co-expression of Pdi and its molecular partner Ero1 was also shown by other authors (Ben Azoun et al., 2016; Sallada et al., 2019).

Thus, addition of known ER network folding factors to enhance heterologous expression has become a basic approach. However, this is not a universal solution; there is no guarantee that chaperones are always able to improve the secretion of recombinant proteins. In particular, a combination of different chaperones does not always lead to a synergistic effect.

*Limitations of the approach*

Recent investigations suggest that the efficiency of chaperones depends on the dose of the gene encoding the recombinant protein. For example, overexpression of Pdi1 or Ero1 caused a statistically significant increase in recombinant hydrophobin in P. pastoris, but only in the 3-copy gene strain, and not in the 1- and 2-copy strains (Sallada et al., 2019). In the same experiment, it was shown that hydrophobin production increased with co-expression of Kar2 14-fold for the 1-copy strain, 9.8-fold for the 2-copy and 22-fold for the 3-copy ones (Sallada et al., 2019).

A correlation between chaperone activity and the copy number of the recombinant gene has been found in other experiments as well. In lipase-producing P. pastoris, overexpression of Pdi1 led to enhanced productivity in the strain carrying four copies of the lipase gene, whereas in the twocopy strain, it remained unchanged (Huang J. et al., 2020). Efficient expression of mammalian peptidoglycan recognition proteins in P. pastoris also depended on the combination of recombinant gene copy number and folding enzymes (Yang et al., 2016). Thus, these experiments support the need for empirical selection of optimal combinations of recombinant protein-encoding genes and folding factor-encoding genes.

Recently, an increasing number of published data have demonstrated that the activity of the folding factors described above is selective for certain substrates and does not improve the expression of some important recombinant proteins. In particular, it was shown that Pdi1, Ero1, Kar2 did not have a beneficial effect on the secretion of antibodies in S. cerevisiae (de Ruijter et al., 2016). Similar conclusions were also drawn from other experiments (Smith et al., 2004; Payne et al., 2008). These findings demonstrate that there is no single suitable strategy that provides optimal conditions for the secretion of all recombinant proteins, and very often a separate productivity enhancement program must be set up for each protein.

*Forced activation of the UPR system*

An effective way to solve the folding problem is the activation of the ER chaperone network by overexpression of the Hac1 transcription factor, which is the main regulating UPR pathway. Theoretically, an artificial increase in the level of Hac1 can be achieved in two ways. The first is the intensification of Hac1 mRNA splicing by overexpression of Ire1. The second way is overexpression of genetic sequences encoding Hac1.

The first approach is associated with additional expression of Ire1, which should lead to an increase in the number of events associated with Hac1 mRNA splicing and, as a consequence, promote transcription of genes encoding ER chaperones and folding catalysts (see above “Unfolded protein response”). However, at present, the effectiveness of this approach is confirmed by only one experiment. Only one study convincingly showed that overexpression of Ire1 improved the capabilities of the yeast expression system, which in particular led to an increase in hepatitis B small antigen (HBsAg) production in S. cerevisiae (Sheng et al., 2017). The lack of positive results may be due to the complex mechanism of Hac1 activation, which involves many molecular factors.

The second approach aimed at using Hac1 overexpression is the most preferable in yeast biotechnology. As we wrote above, adding Hac1 to yeast host strains leads to up-regulation of a large number of its target genes that mainly function for the protein secretory machinery, and in particular, almost all these genes are required for protein folding (see above “Unfolded protein response”). For heterologous protein secretion, both the active (spliced) form and the inactive (unspliced) form of Hac1 can be used. However, activation of the unspliced form of Hac1 mRNA requires additional cellular resources, in particular, molecular factors involved in its processing. The genes encoding these factors must be added to the yeast strains. The engineered strains with such a modification increased protein secretion significantly and showed better performance than strains with only overexpression of Hac1 (Lin et al., 2023).

Most strains used in biotechnological practice contain an insertion of the Hac1 gene with a previously removed 3′ end intron, i. e. encoding the active form of the transcript. For such strains, a high level of transcriptional activity of Hac1 target genes involved in the UPR pathway and responsible for correct protein folding was shown. In P. pastoris strains overexpressing Hac1, electron microscopy revealed an expansion of the intracellular membranes (Guerfal et al., 2010), which probably indicates an increase in the folding capacity of the ER and may contribute to the alleviation of ER stress (Schuck et al., 2009).

To date, a large number of research groups have reported an increase in the productivity of secretory proteins due to artificial and high-level expression of the Hac1 protein (Raschmanová et al., 2021; Lin et al., 2023; Khlebodarova et al., 2024). Hac1 overexpression successfully increased the yield of recombinant proteins: phytase (a product of the Phy gene from C. amalonaticus) in P. pastoris (Li C. et al., 2015); α-amylase (Valkonen et al., 2003; Lin et al., 2023); chitosanase (from Bacillus subtilis) (Han et al., 2021); kringle fragment of human apolipoprotein (which inhibits endothelial cell migration) in S. cerevisiae (Lee et al., 2012); xylanase (a microbial hydrolase used to hydrolyze xylan) in S. cerevisiae (Li C. et al., 2015; Bao et al., 2020).

However, to achieve efficient heterologous expression in Hac-modified cells, it is important to select the optimal combination of the copy number of Hac-encoding genes and genes encoding recombinant proteins (Valkonen et al., 2003; Guerfal et al., 2010; Huang M. et al., 2017; Huang J. et al., 2020). Some studies have found that increasing Hac1 doses also leads to increased ER stress (Gasser et al., 2006; Guerfal et al., 2010; Li C. et al., 2015). It is also necessary to take into account that Hac1 is a positive regulator of the ERAD pathway (see above “ER-associated degradation”) and its overactivation can promote protein degradation.

Thus, the addition of Hac allows the yeast strain to be adapted to large-scale protein expression caused by an excessive dose of transgenic constructs. However, many researchers note that the effectiveness of this approach must be assessed in each specific case when designing a specific producer strain

An additional advantage in strain engineering may be gained by using Hac orthologs from other yeasts, and even phylogenetically more distant eukaryotes. In particular, Valkonen et al. (2003) reported that the secretion of α-amylase was increased by overexpressing Trichoderma reesei-derived Hac1 in S. cerevisiae. Bankefa and colleagues (2018) showed that for P. pastoris, Hac1 orthologs of other species and even mammalian ones may be more effective than the native one. They investigate the effects of overexpressing Hac1 orthologs from S. cerevisiae (ScHac1p), Trichoderma reesei (TrHac1p) and Homo sapiens (HsXbp1) on the secretory expression levels of three reporter proteins, b-galactosidase, b-mannanase and glucose oxidase. The authors reported diverse effects of these orthologs on heterologous expression levels, but HsXbp1 remarkably improved the enzyme production levels, both in shake flask and fermenter culture, both in single- and fourcopy strains, which demonstrated its great application potential (Bankefa et al., 2018).

Thus, artificial activation of the UPR pathway by overexpression of the Hac1 transcription factor has demonstrated an obvious positive effect on improving the secretory protein productivity. It allowed to remove bottlenecks in the engineered yeast strain, arising due to abnormal accumulation of unfolded/misfolded proteins in the endoplasmic reticulum. However, this positive experience cannot be extrapolated to all recombinant proteins. Therefore, the real effect for each product must be assessed in experiments, often based on trial and error.

*Prevention of ERAD pathway activation*

As we wrote above, the UPR system is in crosstalk with the ERAD pathway. ERAD is activated when ER chaperones and folding enzymes are unable to form tertiary or quaternary structures of proteins. Sometimes ERAD is excessively activated during heterologous expression, so depletion of some components of this system can contribute to an increase in the yield of recombinant proteins.

De Ruijter and Frey (2015) analyzed the effect of deletions of genes involved in ERAD on the production of human IgG in S. cerevisiae. It was shown that deletion of only one gene, HTM1, contributed to a slight improvement in heterologous secretion, whereas deletions of the yos9, hrd1, hrd3, and ubc7 genes either did not affect or negatively affected the recombinant protein yield (de Ruijter, Frey , 2015).

In P. pastoris, excessive activation of ERAD enhanced intracellular degradation of recombinant antibody fragment Fab. In the work of Pfeffer et al., it was shown that most of the newly synthesized Fab is not secreted but undergoes intracellular degradation via the ubiquitin-proteasome system (Pfeffer et al., 2012; Zahrl et al., 2019). The yield of the recombinant protein was increased by inhibiting proteasome components (Pfeffer et al., 2012). However, subsequent work aimed at reducing proteolysis through ERAD gene disruptions did not yield significant increases in Fab secretion (Zahrl et al., 2019).

Thus, the reduction of ERAD activity can be considered as a potential strategy for improving the secretion of recombinant proteins. However, the current level of research in this area does not yet allow for the transition to engineering producer strains protected from the proteolysis system

*Search for new solutions*

The potential of the ER folding network can be enhanced by factors that are not directly involved in it, but create favorable conditions for its active functioning.

Zahrl et al. (2023) proposed to combine the transcriptional programs induced by Hac1 and Msn4 in one strain. Msn4 is a transcriptional factor involved in the response to various forms of stress (heat, oxidative, osmotic, etc.). Co-expression of Hac1 and Msn4 (both native and synthetic) revealed synergistic effects resulting in increased titers of recombinant proteins. This strategy was tested for scFv and VHH antibody fragments expressed in P. pastoris (Zahrl et al., 2023).

In another work, the same research group identified the most relevant chaperones of the Hsp70 network, both cytosolic and ER-localized, and investigated the impact of their combined overexpression on recombinant protein secretion (Zahrl et al., 2022). In their work , they implemented a principle they called the push-and-pull strategy. The addition of cytosolic chaperones allowed to increase the translocation competency of the recombinant protein and its targeting to the ER membrane (= push). At the same time resident ER chaperones improved the folding process (= pull). This allowed to successfully engineer strains and improve protein secretion up to 5-fold for the antibody Fab fragment and scFv (Zahrl et al., 2022).

The screening of new molecules involved in the folding system may improve yeast expression systems and expand the range of heterologous proteins. In particular, analysis of the reported P. pastoris secretome and genome predicted novel folding factors: Mpd1 and Pdi2 (members of the Pdi family), as well as Sil1 (nucleotide exchange factor for Kar2) (Duan et al., 2019). Subsequent experimental studies showed that all of the novel folding factors enhanced total production of reporter proteins, with Sil1 showing the highest efficiency (Duan et al., 2019). This work is an example of a successful combination of the achievements of yeast omics technologies and metabolic engineering, but this experience has not yet been widely applied.

## Conclusion

One of the main limitations of heterologous protein production in yeast hosts is the ability of proteins to fold in the endoplasmic reticulum. The folding system is subject to unbalanced stress due to overexpression of recombinant genes, leading to the accumulation of misfolded proteins, aggregate formation, and low productivity. However, thanks to years of effective research into the fundamental mechanisms of protein folding, these limitations have been largely overcome. Studying folding in both model organisms and bioproducers has enabled the identification of molecular factors and cellular mechanisms that determine how a nascent polypeptide chain acquires its three-dimensional functional structure. This knowledge has formed the basis for the development of new, efficient methods for constructing highly productive yeast strains. Many problems arising from insufficient folding systems have been overcome by selecting optimal signal peptides, coexpressing with chaperones and foldases, modifying the ubiquitin-proteasome system (UPS), and preventing the ERAD pathway. Modern engineering solutions utilize combinations of these factors, but for each protein of interest, the expression strain is typically developed individually. In practice, optimized folding conditions for one protein often do not work for another. Therefore, no general strategy for overcoming protein folding bottlenecks that would be applicable to a wide range of proteins has yet been proposed

In the future, some problems can be minimized by analyzing data obtained using omics technologies and modeling the secretion pathway in silico. An example of such a development is the pcSecYeast model designed for S. cerevisiae (Li F. et al., 2022). Such models allow choosing a combination of factors, both known and unknown, to generate new engineering strategies in designing strains with high protein yields.

## Conflict of interest

The authors declare no conflict of interest.

## References

Aza P., Molpeceres G., de Salas F., Camarero S. Design of an improved
universal signal peptide based on the α-factor mating secretion signal
for enzyme production in yeast. Cell Mol Life Sci. 2021;78(7):
3691-3707. doi 10.1007/s00018-021-03793-y

Bankefa O.E., Wang M., Zhu T., Li Y. Hac1p homologues from higher
eukaryotes can improve the secretion of heterologous proteins in the
yeast Pichia pastoris. Biotechnol Lett. 2018;40(7):1149-1156. doi
10.1007/s10529-018-2571-y

Bao C., Li J., Chen H., Sun Y., Wang G., Chen G., Zhang S. Expression
and function of an Hac1-regulated multi-copy xylanase gene in
Saccharomyces cerevisiae. Sci Rep. 2020;10(1):11686. doi 10.1038/
s41598-020-68570-6

Barrero J.J., Casler J.C., Valero F., Ferrer P., Glick B.S. An improved
secretion signal enhances the secretion of model proteins from
Pichia
pastoris. Microb Cell Fact. 2018;17(1):161. doi 10.1186/
s12934-018-1009-5

Beal D.M., Bastow E.L., Staniforth G.L., von der Haar T., Freedman
R.B., Tuite M.F. Quantitative analyses of the yeast oxidative
protein folding pathway in vitro and in vivo. Antioxid Redox Signal.
2019;31(4):261-274. doi 10.1089/ars.2018.7615

Ben Azoun S., Belhaj A.E., Göngrich R., Gasser B., Kallel H. Molecular
optimization of rabies virus glycoprotein expression in Pichia
pastoris. Microb Biotechnol. 2016;9(3):355-368. doi 10.1111/1751-
7915.12350

Berner N., Reutter K.R., Wolf D.H. Protein quality control of the endoplasmic
reticulum and ubiquitin-proteasome-triggered degradation
of aberrant proteins: yeast pioneers the path. Annu Rev Biochem.
2018;20(87):751-782. doi 10.1146/annurev-biochem-062917-012749

Braakman I., Hebert D.N. Protein folding in the endoplasmic reticulum.
Cold Spring Harb Perspect Biol. 2013;5(5):a013201. doi 10.1101/
cshperspect.a013201

Burgess R.R. Refolding solubilized inclusion body proteins. Methods
Enzymol. 2009;463:259-282. doi 10.1016/S0076-6879(09)63017-2

Caramelo J.J., Parodi A.J. A sweet code for glycoprotein folding. FEBS
Lett. 2015;589(22):3379-3387. doi 10.1016/j.febslet.2015.07.021

Damasceno L.M., Anderson K.A., Ritter G., Cregg J.M., Old L.J.,
Batt C.A. Cooverexpression of chaperones for enhanced secretion of
a single-chain antibody fragment in Pichia pastoris. Appl Microbiol
Biotechnol. 2007;74(2):381-389. doi 10.1007/s00253-006-0652-7

De Brabander P., Uitterhaegen E., Delmulle T., De Winter K., Soetaert
W. Challenges and progress towards industrial recombinant
protein production in yeasts: a review. Biotechnol Adv. 2023;64:
108121. doi 10.1016/j.biotechadv.2023.108121

de Keyzer J., Steel G.J., Hale S.J., Humphries D., Stirling C.J.
Nucleotide binding by Lhs1p is essential for its nucleotide exchange
activity and for function in vivo. J Biol Chem. 2009;284(46):31564-
31571. doi 10.1074/jbc.M109.055160

de Ruijter J.C., Frey A.D. Analysis of antibody production in Saccharomyces
cerevisiae: effects of ER protein quality control disruption.
Appl Microbiol Biotechnol. 2015;99:9061-9071. doi 10.1007/
s00253-015-6807-7

de Ruijter J.C., Koskela E.V., Frey A.D. Enhancing antibody folding
and secretion by tailoring the Saccharomyces cerevisiae endoplasmic
reticulum. Microb Cell Fact. 2016;23(15):87. doi 10.1186/
s12934-016-0488-5

Duan G., Ding L., Wei D., Zhou H., Chu J., Zhang S., Qian J. Screening
endogenous signal peptides and protein folding factors to promote
the secretory expression of heterologous proteins in Pichia pastoris.
J Biotechnol. 2019;306:193-202. doi 10.1016/j.jbiotec.2019.06.297

Eskandari A., Nezhad N.G., Leow T.C., Rahman M.B.A., Oslan S.N.
Current achievements, strategies, obstacles, and overcoming the
challenges of the protein engineering in Pichia pastoris expression
system. World J Microbiol Biotechnol. 2023;40(1):39. doi 10.1007/
s11274-023-03851-6

Fauzee Y.N.B.M., Taniguchi N., Ishiwata-Kimata Y., Takagi H., Kimata
Y. The unfolded protein response in Pichia pastoris without
external stressing stimuli. FEMS Yeast Res. 2020;20(7):foaa053. doi
10.1093/femsyr/foaa053

Frand A.R., Kaiser C.A. The Ero1 gene of yeast is required for oxidation
of protein dithiols in the endoplasmic reticulum. Mol Cell.
1998;1(2):161-170. doi 10.1016/s1097-2765(00)80017-9

Friedlander R., Jarosch E., Urban J., Volkwein C., Sommer T. A regulatory
link between ER-associated protein degradation and the
unfolded-protein response. Nat Cell Biol. 2000;2(7):379-384. doi
10.1038/35017001Gasser B., Maurer M., Gach J., Kunert R., Mattanovich D. Engineering
of Pichia pastoris for improved production of antibody fragments.
Biotechnol Bioeng. 2006;94(2):353-361. doi 10.1002/bit.20851

Gasser B., Saloheimo M., Rinas U., Dragosits M., Rodríguez-Carmona
E., Baumann K., Giuliani M., … Porro D., Ferrer P., Tutino
M.L., Mattanovich D., Villaverde A. Protein folding and conformational
stress in microbial cells producing recombinant proteins:
a host comparative overview. Microb Cell Fact. 2008;4(7):11. doi
10.1186/1475-2859-7-11

Gross E., Sevier C.S., Heldman N., Vitu E., Bentzur M., Kaiser C.A.,
Thorpe C., Fass D. Generating disulfides enzymatically: reaction
products and electron acceptors of the endoplasmic reticulum thiol
oxidase Ero1p. Proc Natl Acad Sci USA. 2006;103(2):299-304. doi
10.1073/pnas.0506448103

Guerfal M., Ryckaert S., Jacobs P.P., Ameloot P., Van Craenenbroeck
K., Derycke R., Callewaert N. The HAC1 gene from Pichia
pastoris: characterization and effect of its overexpression on the
production of secreted, surface displayed and membrane proteins.
Microb Cell Fact. 2010;9:49-60. doi 10.1186/1475-2859-9-49

Han M., Wang W., Gong X., Zhou J., Xu C., Li Y. Increased expression
of recombinant chitosanase by co-expression of Hac1p in the yeast
Pichia pastoris. Protein Pept Lett. 2021;28(12):1434-1441. doi
10.2174/0929866528666211105111155

Hartl F.U., Bracher A., Hayer-Hartl M. Molecular chaperones in protein
folding and proteostasis. Nature. 2011;475(7356):324-332. doi
10.1038/nature10317

Hatahet F., Ruddock L.W. Protein disulfide isomerase: a critical evaluation
of its function in disulfide bond formation. Antioxid Redox Signal.
2009;11(11):2807-2850. doi 10.1089/ars.2009.2466

Hendershot L.M., Buck T.M., Brodsky J.L. The essential functions of
molecular chaperones and folding enzymes in maintaining endoplasmic
reticulum homeostasis. J Mol Biol. 2024;436(14):168418. doi
10.1016/j.jmb.2023.168418

Hernández-Elvira M., Torres-Quiroz F., Escamilla-Ayala A., Domínguez-
Martin E., Escalante R., Kawasaki L., Ongay-Larios L., Coria
R. The unfolded protein response pathway in the yeast Kluyveromyces
lactis. A comparative view among yeast species. Cells.
2018;7(8):106. doi 10.3390/cells7080106

Herscovics A. Processing glycosidases of Saccharomyces cerevisiae.
Biochim Biophys Acta. 1999;1426(2):275-285. doi 10.1016/s0304-
4165(98)00129-9

Huang J., Zhao Q., Chen L., Zhang C., Bu W., Zhang X., Zhang K.,
Yang Z. Improved production of recombinant Rhizomucor miehei lipase
by coexpressing protein folding chaperones in Pichia pastoris,
which triggered ER stress. Bioengineered. 2020;11(1):375-385. doi
10.1080/21655979.2020.1738127

Huang M., Gao Y., Zhou X., Zhang Y., Cai M. Regulating unfolded
protein response activator HAC1p for production of thermostable
raw-starch hydrolyzing α-amylase in Pichia pastoris. Bioprocess
Biosyst Eng. 2017;40(3):341-350. doi 10.1007/s00449-016-1701-y

Inan M., Fanders S.A., Zhang W., Hotez P.J., Zhan B., Meagher M.M.
Saturation of the secretory pathway by overexpression of a hookworm
(Necator americanus) protein (Na-ASP1). In: Cregg J.M.
(Ed.) Pichia Protocols. Methods in Molecular Biology. Vol. 389.
Humana Press, 2007;65-76. doi 10.1007/978-1-59745-456-8_5

Ishiwata-Kimata Y., Kimata Y. Fundamental and applicative aspects
of the unfolded protein response in yeasts. J Fungi (Basel). 2023;
9(10):989. doi 10.3390/jof9100989

Ito Y., Ishigami M., Hashiba N., Nakamura Y., Terai G., Hasunuma T.,
Ishii J., Kondo A. Avoiding entry into intracellular protein degradation
pathways by signal mutations increases protein secretion
in Pichia pastoris. Microb Biotechnol. 2022;15(9):2364-2378. doi
10.1111/1751-7915.14061

Kelleher D.J., Gilmore R. An evolving view of the eukaryotic oligosaccharyltransferase.
Glycobiology. 2006;16(4):47R-62R. doi 10.1093/
glycob/cwj066

Khlebodarova T.M., Bogacheva N.V., Zadorozhny A.V., Bryanskaya
A.V., Vasilieva A.R., Chesnokov D.O., Pavlova E.I., Peltek
S.E. Komagataella phaffii as a platform for heterologous expression
of enzymes used for industry. Microorganisms. 2024;12(2):
346. doi 10.3390/microorganisms12020346

Kim M.D., Han K.C., Kang H.A., Rhee S.K., Seo J.H. Coexpression
of BiP increased antithrombotic hirudin production in recombinant
Saccharomyces cerevisiae. J Biotechnol. 2003;101(1):81-87. doi
10.1016/s0168-1656(02)00288-2

Kimmig P., Diaz M., Zheng J., Williams C.C., Lang A., Aragón T.,
Li H., Walter P. The unfolded protein response in fission yeast modulates
stability of select mRNAs to maintain protein homeostasis.
eLife. 2012;1:e00048. doi 10.7554/eLife.00048

Korennykh A., Walter P. Structural basis of the unfolded protein response.
Annu Rev Cell Dev Biol. 2012;28:251-277. doi 10.1146/
annurev-cellbio-101011-155826

Krshnan L., van de Weijer M.L., Carvalho P. Endoplasmic reticulumassociated
protein degradation. Cold Spring Harb Perspect Biol.
2022;14(12):a041247. doi 10.1101/cshperspect.a041247

Lee T.H., Bae Y.H., Kim M.D., Seo J.H. Overexpression of Hac1 gene
increased levels of both intracellular and secreted human kringle
fragment in Saccharomyces cerevisiae. Process Biochem. 2012;
47(12):2300-2305. doi 10.1016/j.procbio.2012.09.006

Lehle L., Strahl S., Tanner W. Protein glycosylation, conserved from
yeast to man: a model organism helps elucidate congenital human
diseases. Angew Chem Int Ed Engl. 2006;45(41):6802-6818. doi
10.1002/anie.200601645

Li C., Lin Y., Zheng X., Pang N., Liao X., Liu X., Huang Y., Liang S.
Combined strategies for improving expression of Citrobacter amalonaticus
phytase in Pichia pastoris. BMC Biotechnol. 2015;15:88.
doi 10.1186/s12896-015-0204-2

Li F., Chen Y., Qi Q., Wang Y., Yuan L., Huang M., Elsemman I.E.,
Feizi A., Kerkhoven E.J., Nielsen J. Improving recombinant protein
production by yeast through genome-scale modeling using proteome
constraints. Nat Commun. 2022;13(1):2969. doi 10.1038/s41467-
022-30689-7

Lin Y., Feng Y., Zheng L., Zhao M., Huang M. Improved protein production
in yeast using cell engineering with genes related to a key
factor in the unfolded protein response. Metab Eng. 2023;77:152-
161. doi 10.1016/j.ymben.2023.04.004

Madhavan A., Arun K.B., Sindhu R., Krishnamoorthy J., Reshmy R.,
Sirohi R., Pugazhendi A., Awasthi M.K., Szakacs G., Binod P. Customized
yeast cell factories for biopharmaceuticals: from cell engineering
to process scale up. Microb Cell Fact. 2021;20(1):124. doi
10.1186/s12934-021-01617-z

Mizunaga T., Katakura Y., Miura T., Maruyama Y. Purification and
characterization of yeast protein disulfide isomerase. J Biochem.
1990;108(5):846-851. doi 10.1093/oxfordjournals.jbchem.a123291

Mori A., Hara S., Sugahara T., Kojima T., Iwasaki Y., Kawarasaki Y.,
Sahara T., Ohgiya S., Nakano H. Signal peptide optimization tool
for the secretion of recombinant protein from Saccharomyces cerevisiae.
J Biosci Bioeng. 2015;120(5):518-525. doi 10.1016/j.jbiosc.
2015.03.003

Mori K. Evolutionary aspects of the unfolded protein response. Cold
Spring Harb Perspect Biol. 2022;14(12):a041262. doi 10.1101/
cshperspect.a041262

Niu Y., Zhang L., Yu J., Wang C.C., Wang L. Novel roles of the noncatalytic
elements of yeast protein-disulfide isomerase in its interplay
with endoplasmic reticulum oxidoreductin 1. J Biol Chem.
2016;291(15):8283-8294. doi 10.1074/jbc.M115.694257

Núñez A., Dulude D., Jbel M., Rokeach L.A. Calnexin is essential for
survival under nitrogen starvation and stationary phase in Schizosaccharomyces
pombe. PLoS One. 2015;10(3):e0121059. doi 10.1371/
journal.pone.0121059

O’Keefe S., Pool M.R., High S. Membrane protein biogenesis at the
ER: the highways and byways. FEBS J. 2022;289(22):6835-6862.
doi 10.1111/febs.15905

Omkar S., Mitchem M.M., Hoskins J.R., Shrader C., Kline J.T., Nitika,
Fornelli L., Wickner S., Truman A.W. Acetylation of the yeast
Hsp40 chaperone protein Ydj1 fine-tunes proteostasis and translational
fidelity. PLoS Genet. 2024;20(12):e1011338. doi 10.1371/
journal.pgen.1011338

Palma A., Rettenbacher L.A., Moilanen A., Saaranen M., Pacheco-
Martinez C., Gasser B., Ruddock L. Biochemical analysis of Komagataella
phaffii oxidative folding proposes novel regulatory mechanisms
of disulfide bond formation in yeast. Sci Rep. 2023;13(1):
14298. doi 10.1038/s41598-023-41375-z

Parlati F., Dominguez M., Bergeron J.J., Thomas D.Y. Saccharomyces
cerevisiae CNE1 encodes an endoplasmic reticulum (ER) membrane
protein with sequence similarity to calnexin and calreticulin and
functions as a constituent of the ER quality control apparatus. J Biol
Chem. 1995;270(1):244-253. doi 10.1074/jbc.270.1.244

Payne T., Finnis C., Evans L.R., Mead D.J., Avery S.V., Archer D.B.,
Sleep D. Modulation of chaperone gene expression in mutagenized
Saccharomyces cerevisiae strains developed for recombinant human
albumin production results in increased production of multiple
heterologous proteins. Appl Environ Microbiol. 2008;74(24):7759-
7766. doi 10.1128/AEM.01178-08

Pfeffer M., Maurer M., Stadlmann J., Grass J., Delic M., Altmann F.,
Mattanovich D. Intracellular interactome of secreted antibody Fab
fragment in Pichia pastoris reveals its routes of secretion and degradation.
Appl Microbiol Biotechnol. 2012;93(6):2503-2512. doi
10.1007/s00253-012-3933-3

Pickart C.M. Mechanisms underlying ubiquitination. Annu Rev Biochem.
2001;70:503-533. doi 10.1146/annurev.biochem.70.1.503

Preston G.M., Brodsky J.L. The evolving role of ubiquitin modification
in endoplasmic reticulum-associated degradation. Biochem J.
2017;474(4):445-469. doi 10.1042/BCJ20160582

Qi Q., Li F., Yu R., Engqvist M.K.M., Siewers V., Fuchs J., Nielsen J.
Different routes of protein folding contribute to improved protein
production in Saccharomyces cerevisiae. mBio. 2020;11(6):
e02743-20. doi 10.1128/mBio.02743-20

Raschmanová H., Weninger A., Knejzlík Z., Melzoch K., Kovar K.
Engineering of the unfolded protein response pathway in Pichia
pastoris: enhancing production of secreted recombinant proteins.
Appl Microbiol Biotechnol. 2021;105(11):4397-4414. doi 10.1007/
s00253-021-11336-5

Robinson A.S., Hines V., Wittrup K.D. Protein disulfide isomerase
overexpression increases secretion of foreign proteins in Saccharomyces
cerevisiae. Nat Biotechnol. 1994;12(4):381-384. doi 10.1038/
nbt0494-381

Ruger-Herreros C., Svoboda L., Mogk A., Bukau B. Role of J-domain
proteins in yeast physiology and protein quality control. J Mol Biol.
2024;436(14):168484. doi 10.1016/j.jmb.2024.168484

Ruggiano A., Foresti O., Carvalho P. Quality control: ER-associated
degradation: protein quality control and beyond. J Cell Biol. 2014;
204(6):869-879. doi 10.1083/jcb.201312042

Saibil H. Chaperone machines for protein folding, unfolding and disaggregation.
Nat Rev Mol Cell Biol. 2013;14(10):630-642. doi
10.1038/nrm3658

Sallada N.D., Harkins L.E., Berger B.W. Effect of gene copy number
and chaperone coexpression on recombinant hydrophobin HFBI
biosurfactant production in Pichia pastoris. Biotechnol Bioeng.
2019;116(8):2029-2040. doi 10.1002/bit.26982

Schlenstedt G., Harris S., Risse B., Lill R., Silver P.A. A yeast DnaJ
homologue, Scj1p, can function in the endoplasmic reticulum with BiP/Kar2p via a conserved domain that specifies interactions with
Hsp70s. J Cell Biol. 1995;129(4):979-988. doi 10.1083/jcb.129.
4.979

Schroder M., Clark R., Kaufman R.J. IRE1- and HAC1-independent
transcriptional regulation in the unfolded protein response of yeast.
Mol Microbiol. 2003;49(3):591-606. doi 10.1046/j.1365-2958.2003.
03585.x

Schuck S., Prinz W.A., Thorn K.S., Voss C., Walter P. Membrane expansion
alleviates endoplasmic reticulum stress independently of
the unfolded protein response. J Cell Biol. 2009;187(4):525-536. doi
10.1083/jcb.200907074

Schwarz D.S., Blower M.D. The endoplasmic reticulum: structure,
function and response to cellular signaling. Cell Mol Life Sci. 2016;
73(1):79-94. doi 10.1007/s00018-015-2052-6

Sevier C.S., Kaiser C.A. Disulfide transfer between two conserved cysteine
pairs imparts selectivity to protein oxidation by Ero1. Mol Biol
Cell. 2006;17(5):2256-2266. doi 10.1091/mbc.e05-05-0417

Sheng J., Flick H., Feng X. Systematic optimization of protein secretory
pathways in Saccharomyces cerevisiae to increase expression of
hepatitis B small antigen. Front Microbiol. 2017;8:875. doi 10.3389/
fmicb.2017.00875

Shusta E.V., Raines R.T., Plückthun A., Wittrup K.D. Increasing the
secretory capacity of Saccharomyces cerevisiae for production of
single-chain antibody fragments. Nat Biotechnol. 1998;16(8):773-
777. doi 10.1038/nbt0898-773

Singhvi P., Saneja A., Ahuja R., Panda A.K. Solubilization and refolding
of variety of inclusion body proteins using a novel formulation.
Int J Biol Macromol. 2021;193(Pt.B):2352-2364. doi 10.1016/
j.ijbiomac.2021.11.068

Smith J.D., Tang B.C., Robinson A.S. Protein disulfide isomerase, but
not binding protein, overexpression enhances secretion of a nondisulfide-
bonded protein in yeast. Biotechnol Bioeng. 2004;85(3):
340-350. doi 10.1002/bit.10853

Steel G.J., Fullerton D.M., Tyson J.R., Stirling C.J. Coordinated activation
of Hsp70 chaperones. Science. 2004;303(5654):98-101. doi
10.1126/science.1092287

Thak E.J., Yoo S.J., Moon H.Y., Kang H.A. Yeast synthetic biology
for designed cell factories producing secretory recombinant proteins.
FEMS Yeast Res. 2020;20(2):foaa009. doi 10.1093/femsyr/
foaa009

Thibault G., Ng D.T.W. The endoplasmic reticulum-associated degradation
pathways of budding yeast. Cold Spring Harb Perspect Biol.
2012;4(12):a013193. doi 10.1101/cshperspect.a013193

Travers K.J., Patil C.K., Wodicka L., Lockhart D.J., Weissman J.S.,
Walter P. Functional and genomic analyses reveal an essential coordination
between the unfolded protein response and ER-associated
degradation. Cell. 2000;101(3):249-258. doi 10.1016/s0092-
8674(00)80835-1

Tsuda M., Nonaka K. Recent progress on heterologous protein production
in methylotrophic yeast systems. World J Microbiol Biotechnol.
2024;40(7):200. doi 10.1007/s11274-024-04008-9

Valkonen M., Penttila M., Saloheimo M. Effects of inactivation and
constitutive expression of the unfolded-protein response pathway
on protein production in the yeast Saccharomyces cerevisiae. Appl
Environ Microbiol. 2003;69(4):2065-2072. doi 10.1128/aem.69.4.
2065-2072.2003

Wang L., Wang C.C. Oxidative protein folding fidelity and redoxtasis in
the endoplasmic reticulum. Trends Biochem Sci. 2023;48(1):40-52.
doi 10.1016/j.tibs.2022.06.011

Ware F.E., Vassilakos A., Peterson P.A., Jackson M.R., Lehrman
M.A., Williams D.B. The molecular chaperone calnexin binds
Glc1Man9GlcNAc2 oligosaccharide as an initial step in recognizing
unfolded glycoproteins. J Biol Chem. 1995;270(9):4697-4704. doi
10.1074/jbc.270.9.4697

Xia X. Translation control of HAC1 by regulation of splicing in Saccharomyces
cerevisiae. Int J Mol Sci. 2019;20(12):2860. doi 10.3390/
ijms20122860

Xu C., Ng D.T.W. Glycosylation-directed quality control of protein
folding. Nat Rev Mol Cell Biol. 2015;16:742-752. doi 10.1038/
nrm4073

Xu X., Kanbara K., Azakam H., Kato A. Expression and characterization
of Saccharomyces cerevisiae Cne1p, a calnexin homologue.
J Biochem. 2004;135(5):615-618. doi 10.1093/jb/mvh074

Yamaguchi H., Miyazaki M. Refolding techniques for recovering biologically
active recombinant proteins from inclusion bodies. Biomolecules.
2014;4(1):235-251. doi 10.3390/biom4010235

Yang J., Lu Z., Chen J., Chu P., Cheng Q., Liu J., Ming F., Huang C.,
Xiao A., Cai H., Zhang L. Effect of cooperation of chaperones and
gene dosage on the expression of porcine PGLYRP-1 in Pichia
pastoris. Appl Microbiol Biotechnol. 2016;100(12):5453-5465. doi
10.1007/s00253-016-7372-4

Zahrl R.J., Gasser B., Mattanovich D., Ferrer P. Detection and elimination
of cellular bottlenecks in protein-producing yeasts. In: Gasser
B., Mattanovich D. (Eds) Recombinant Protein Production in
Yeast. Methods in Molecular Biology. Vol. 1923. Humana Press,
2019;75-95. doi 10.1007/978-1-4939-9024-5_2

Zahrl R.J., Prielhofer R., Ata Ö., Baumann K., Mattanovich D., Gasser
B. Pushing and pulling proteins into the yeast secretory pathway
enhances recombinant protein secretion. Metab Eng. 2022;74:
36-48. doi 10.1016/j.ymben.2022.08.010

Zahrl R.J., Prielhofer R., Burgard J., Mattanovich D., Gasser B. Synthetic
activation of yeast stress response improves secretion of recombinant
proteins. N Biotechnol. 2023;73:19-28. doi 10.1016/j.nbt.
2023.01.001

Zha J., Liu D., Ren J., Liu Z., Wu X. Advances in metabolic engineering
of Pichia pastoris strains as powerful cell factories. J Fungi
(Basel).
2023;9(10):1027. doi 10.3390/jof9101027

